# Medical Benefits of Honeybee Products

**DOI:** 10.1155/2017/2702106

**Published:** 2017-05-09

**Authors:** José Maurício Sforcin, Vassya Bankova, Andrzej K. Kuropatnicki

**Affiliations:** ^1^Department of Microbiology and Immunology, Biosciences Institute, UNESP, 18618-970 Botucatu, SP, Brazil; ^2^Institute of Organic Chemistry, Centre of Phytochemistry, Bulgarian Academy of Sciences, 1113 Sofia, Bulgaria; ^3^History and Material Culture of English Speaking Countries, Pedagogical University of Krakow, 31-128 Krakow, Poland

The present Special Issue on medical benefits of honeybee products is dedicated to the memory of Professor Wojciech Krol (1956–2016) who was its initiator and first Lead Guest Editor. He specialized in flavons and flavonoids and his interests included propolis and other natural products. Through his studies of propolis, Professor Krol proved the anti-oxdative properties and possibilities of free radicals scavenging through ethanol extract of propolis (EEP) as well as the synergic antibacterial properties of streptomycin and coloxacine together with EEP. Professor W. Krol was also the Lead Guest Editor of the Special Issue “Propolis: Properties, Application, and Its Potential” published in 2013.

Honeybee products have a long medicinal history. All cultures have folk medicine traditions that include the use of honeybee products, that is, honey, bee pollen, propolis, royal jelly, beeswax, and bee venom. These products have been found to exhibit anti-inflammatory, anti-bacterial, anti-fungal, anti-viral and antioxidant activities. It has been also shown that natural honeybee products inhibit tumor cell growth and metastasis and induce apoptosis of cancer cells. Hence, these bioactive natural products may prove to be useful in cancer therapy.

For this special issue we invited researchers and scholars to submit original research reports and review articles in which they explore aspects of the biological activity of a wide range of honeybee products and their possible applications. A total of 46 papers were submitted out of which, after a rigorous peer-review process, 18 manuscripts have been selected because they represent rich and comprehensive new knowledge. Most of the articles in this special issue are of research character and they present the results of a variety of studies comprising different honeybee products. The accepted papers come from Malaysia, Korea, Lithuania, Chile, Japan, Bangladesh, Saudi Arabia, Egypt, and Poland.

Propolis seems to be the most popular bee product, however highly diversified and chemically complex. One of the articles compares chemical composition and biological activity of yellow, green, brown and red Brazilian propolis. The authors conclude that it is difficult to establish a uniform quality standard for propolis form diverse geographical regions. Some authors show that propolis exhibits anti-diabetic activity and reduces the oxidative stress caused by diabetes. Propolis also plays a very important role in lipid metabolism since its use has positive effect on oxidative status and improvement of HDL-c, contributing to a reduced risk of cardiovascular disease. Antioxidant properties and cardioprotective mechanism of propolis from Malaysia were confirmed in rats.

Various articles deal with separate honeybee products. Bee pollen ointment may be effectively used in burn wound treatment since bee pollen prevents infection of the newly-formed tissue in the healing process of burn wounds. Manuka honey tested on rats proved to possess anti-oxidant and anti-inflammatory activities and seems to be effective in the treatment of chronic ulcer and prevention of mucosal glycoproteins. Another study conducted on rats showed that the consumption of Malaysian honey reduces excessive weight gain and improves obesity-related parameters in obese rats. Bee venom, a honeybee product used in traditional folk medicine in Korea and China, finds its application in the treatment of arthritis, cancer and inflammation. Some authors prove that bee venom exerts its anti-inflammatory activity through the IRAK1/TAK1/NF-*κ*B signaling pathway.

Oral medicine is another field in which honeybee products are used and their effectiveness was studied. Honey, the best known bee product, can be used in children suffering from oral mucositis. Topical application of honey is recommended in prophylaxis and treatment of mucositis induced by chemo- and radiotherapy. Brazilian propolis may improve poor oral health in patients with cleft lip and cleft lip and palate treated with orthodontic appliances. Toothpaste containing EEP and tea tree oil has been shown to improve hygiene and periodontium condition in patients treated with acrylic partial dentures.

Finally, one article deals with ethnomedicinal uses of honeybee products. Ethnomedicine has evolved over the millennia of human existence but in the last decade the research interests in ethnomedicine, ethnodentistry as well as ethnopharmacy have increased tremendously. The authors analyzed the archival sources concerning honeybee products used for medicinal purposes over the period of more than 100 years.

